# Eco-friendly synthesis, physicochemical studies, biological assay and molecular docking of steroidal oxime-ethers

**DOI:** 10.17179/excli2014-675

**Published:** 2015-03-02

**Authors:** Mahboob Alam, Dong-Ung Lee

**Affiliations:** 1 Division of Bioscience, Dongguk University, Gyeongju 780-714, Republic of Korea

**Keywords:** biological assay, physicochemical studies, microwave irradiation, steroidal oxime-ethers, molecular docking

## Abstract

The aim of this study was to report the synthesis of biologically active compounds; 7-(2′-aminoethoxyimino)-cholest-5-ene (**4**), a steroidal oxime-ether and its derivatives (**5**,** 6**) *via* a facile microwave assisted solvent free reaction methodology. This new synthetic, eco-friendly, sustainable protocol resulted in a remarkable improvement in the synthetic efficiency (85-93 % yield) and high purity using basic alumina. The synthesized compounds were screened for their antibacterial against six bacterial strains by disc diffusion method and antioxidant potential by DPPH assay. The binding capabilities of a compound **6 **exhibiting good antibacterial potential were assessed on the basis of molecular docking studies and four types of three-dimensional molecular field descriptors. Moreover the structure-antimicrobial activity relationships were studied using some physicochemical and quantum-chemical parameters with GAMESS interface as well as WebMO Job Manager by using the basic level of theory. Hence, this synthetic approach is believed to provide a better scope for the synthesis of steroidal oxime-ether analogues and will be a more practical alternative to the presently existing procedures. Moreover, detailed *in silico* docking studies suggested the plausible mechanism of steroidal oxime-ethers as effective antimicrobial agents.

## Introduction

Solid state synthesis is useful where neither a solvent medium, nor vapor-phase interactions are utilized. This clean and green methodology can be applied virtually using a microwave irradiation. Continuing our strategy of eco-friendly synthesis of steroids (Alam et al., 2012[4]), we herein report the solid state synthesis of steroidal oxime-ethers (**4**-**6**) using basic alumina in dry media employing microwave irradiation for the development of an economical, rapid, and safe method devoid of solvent usage, followed by screening for possible antibacterial activity. The *in silico* screening of steroidal oxime-ethers was carried out in order to ascertain the probable active centers. Novel steroidal derivatives with unexpected applications can be obtained by incorporating/ altering one or more hetero atoms in the steroidal moiety (Banday et al., 2010[5]). Using this method, the structure-activity relationship (SAR) of steroidal oximes as cytotoxic agents are well documented (Krstica et al., 2007[18]; Poza et al., 2007[21]). In addition, cytotoxicities of *O*-alkylated derivatives of 7-oximino-5-androstene and the imidazolyl-substituted steroidal oxime-ethers (Bansal et al., 2010[6]), and 2-alkylaminoethyl analogues of various steroidal oximes (Jindal et al., 2003[16]) have been reported. Some oxime-ethers of cholesterol have been investigated for their anti-bacterial activity (Khan et al., 2012[17]). Synthetic steroidal dioxime-ethers exhibited neuromuscular blocking activity (Garcia et al., 1980[14]). However, some new steroidal oxime-ethers (ω-hydroxyalkyl oximes) (Slavikova et al., 1998[25]), aminoethoxy oximes (Shamsuzzaman et al., 2003[22]; Sharma et al., 2011[23]) have also been synthesized without assay of any biological activities. It has been found that the cytotoxicity of substituted hydroximino steroid is governed by the position of the hydroximino group as well as the nature of the side chain in the steroidal moiety (Cui et al., 2009[11]). However, to the best of our knowledge, there are neither reports about antibacterial/ antioxidant activity nor any information of steroidal oxime-ether synthesis under microwave irradiation conditions. Moreover, the physicochemical and quantum-chemical parameters including SAR of steroidal oxime-ethers with respect to antimicrobial activity has not been well explored. The characterization of compound **4 **and the identity of the compounds (**5** and **6**) has been ascertained by their physical, analytical, spectral data and comparison with literature data, respectively. The *in vitro* biological activity, the subsequent molecular docking studies and 'field points' as extrema of electrostatic, steric, and hydrophobic fields of the steroidal oxime-ethers have also been carried out. These field points are used to define the properties necessary for a molecule to bind in a characteristic way into a specified active site. The hypothesis is that compounds showing a similar field point pattern are likely to bind at the same target site, regardless of structure (Cheeseright et al., 2006[8]). Finally, the quantum-chemical and physicochemical calculations were carried out by DFT at basic level theory and Molinspiration software to study the relationship between the electronic properties and physicochemical parameters with antimicrobial activity of steroidal oxime-ethers analogues, respectively.

## Material and Methods

### Chemistry

Unless otherwise stated, all reagents including chloroethylamine hydrochloride and solvents were purchased from standard sources and used without further purification. Melting points were determined on a Kofler apparatus (Reichert, Vienna, Austria) and were uncorrected. The IR spectra were recorded on KBr pellets with a Spectro Lab-Interspec 2020 FTIR spectrometer (Newbury, UK), and values are given in cm^−1^. ^1^H and ^13^CNMR spectra were run in CDCl_3_ on a Bruker Avance (400 and 100 MHz FT NMR) instrument (Bruker, Rheinstetten Germany) with TMS as an internal standard, and values are given in ppm (δ). Mass spectra were recorded on a JEOL DX-303 mass spectrometer (Jeol Ltd, Akishima, Tokyo, Japan). Elemental analyzers (C, H, N) were conducted using a Carlo Erba analyzer model 1108 (Carlo Erba, Stanford, Canada). Purity as well as the progress of the reaction was checked on pre-coated TLC plates (Merck silica gel 60, F_254_). TLC plates were visualized with UV light and/or in an iodine chamber. Sodium sulfate (anhydrous) was used as a drying agent. Microwave-promoted reactions were carried out using a Laboratory Grade Microwave oven fixed with a temperature monitoring sensor. Steroidal keto-oximes (1-3) were prepared according to published methods (Shamsuzzaman et al., 2003[22]; Soppee et al., 1964[24]).

#### Synthesis of steroidal oxime-ethers (4-6)

**Figure 1:**
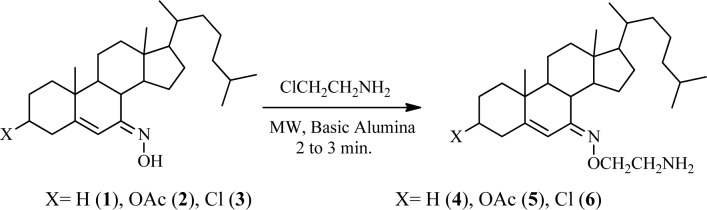
Solid state synthesis of steroidal oxime-ethers 4-6

A mixture of steroidal keto-oxime (1, 2, or 3) (1 mmol), chloroethylamine hydrochloride (1.2 mmol), and basic alumina (2.0 g) was grounded thoroughly with mortar. This reaction mixture was transferred in a glass vessel. It was kept in a microwave oven and irradiated for an appropriate time of 2 to 3 min. After completion of the reaction which was monitored by TLC. It was cooled and then ether (25 mL) was added to the mixture and the alumina was removed by filtration. The filtrate was washed with water, and dried over anhydrous sodium sulfate; evaporation of solvent and crystallization from dry methanol afforded compounds 4-6, respectively (Figure 1(Fig. 1)).

(*Z*)-7-(2′-Aminoethoxyimino)-cholest-5-ene (**4**): mp 163 ºC; IR (KBr) cm^-1^: 3475 (NH_2_), 1665 (C=N), 1405 (N-O) and 1235 (C-N); ^1^H NMR (400 MHz, CDCl_3_): *δ* 5.91 (s, 1H, H-6), 3.31 (m, 2H, NCH_2_), 3.80 (t, *J* = 5.9 Hz, 2H, OCH_2_), 2.7 (s, 2H, NH_2_), 1.11 (s, 3H, C10-CH_3_), 0.71 (s, 3H, C13-CH_3_), 0.91 (d, *J* = 6.2 Hz, 3H C20-CH_3_), 0.86 (d, *J* = 6.8 Hz, 6H, C25-2xCH_3_); ^13^C NMR (100 MHz, CDCl_3_): *δ* 169.1 (C7), 153.3 (C5), 113.9 (C6), 69.3 (OCH_2_), 55.9 (NCH_2_), 53.9 (C17), 51.1 (C14), 50.3 (C13), 43.1 (C9), 38.9 (C10), 38.1 (C8), 37.9 (C24), 37.6 (C12), 37.4 (C4), 37.1 (C1), 36.5 (C22), 35.9 (C20), 31.9 (C3), 27.9 (C16), 27.5 (C25), 27.2 (C2), 26.9 (C15), 24.1 (C23), 23.1 (C26), 22.9 (C27), 21.1 (C11), 19.1 (C21), 18.1 (C19), 13.1 (C18); EI-MS (*m/z*): 443 [M+1]^+•^, 382 [M-OCH_2_CH_2_NH_2_]^+^, 329 [M-C_8_H_17_]^+^.

(*Z*)-3β-Acetoxy-7-(2′-aminoethoxyimino)- cholest-5-ene (**5**): mp 150-151 ºC; IR and ^1^H NMR: see ref. (Shamsuzzaman et al., 2003[22]); ^13^C NMR (100 MHz, CDCl_3_): *δ* 170.1, (OCOCH_3_), 168.3 (C7), 153.9 (C5), 114.1 (C6), 71.0 (C3), 69.7 (OCH_2_), 55.6 (NCH_2_), and other ^13^C NMR signals are in close accordance with those of compound **4**. EI-MS (*m/z*): 500 [M]^+•^, 441 [M-OCOCH_3_]^+^, 440 [M-OCH_2_CH_2_NH_2_]^+^, 387 [M-C_8_H_17_]^+^. 

(*Z*)-3β-Chloro-7-(2′-aminoethoxyimino)-cholest-5-ene (**6**): mp 136-137 ºC; IR and ^1^H NMR: see ref. (Shamsuzzaman et al., 2003[22]); ^13^C NMR (100 MHz, CDCl_3_): *δ* 168.7 (C7), 154.1 (C5), 114.3 (C6), 59.9 (C3), 69.4 (OCH_2_), 55.3 (NCH_2_), and other ^13^C NMR signals are identical to those of compound **4**. EI-MS (*m/z*): 476/478 [M]^+•^, 441 [M-Cl]^+^, 416/418 [M-OCH_2_CH_2_NH_2_]^+^, 363/365 [M-C_8_H_17_]^+^

### Antibacterial activity

Screening for antibacterial is carried out using sterilized antibiotic discs (6 mm), following the standards for Antimicrobial Disc Susceptibility Tests, outlined by the National Committee for Clinical Laboratory Standards-NCCLS (NCCLS, 1993[20]; Collins et al., 1989[10]). The density of the bacterial suspension was standardized by using a McFarland standard method. According to this standard protocol all the assays are carried out at 28 ± 3 °C. Bioassay of the synthesized compounds **4**-**6** were evaluated using the bacterial cultures of the gram negative bacteria *Escherichia coli* (ATCC-25922), *Pseudomonas aeruginosa* (ATCC-27853), and *Klebsiella pneumonia* (ATCC-700603) and the gram positive bacteria *Bacillus subtilis* (ATCC-6051), *Streptococcus pyogenes* (ATCC-29213), and *Staphylococcus aureus* (ATCC-25923) by the disc diffusion method (White and Coon, 1980[28]). Standard reference antibiotic, chloramphenicol, was used as a positive control for the tested bacteria, whereas DMSO was used as a negative control. 

In this method, petri-plates were poured with liquefied agar medium having uniform thickness. After solidification plates were inoculated with test micro-organisms, after which filter paper discs dipped in the test compound solution in DMSO and standard drug solution in DMSO (each 10 μg/mL) were placed in each quadrant of the plate. The drug diffused into the agar medium prevented the growth of microbes and produced a clear zone of inhibition. Plates were first kept at 4 °C for 2 h to allow for diffusion of chemicals, followed by incubation at 28 °C. Antibacterial activity was evaluated by measuring the diameters (mm) of the zones of inhibition in Table 1(Tab. 1) against the tested bacteria after 24 h of incubation. Each assay was replicated in triplicate with SD.

### Antioxidant activity

The free radical scavenging activity of compounds was evaluated by DPPH assay using the reported protocol (Blois, 1958[7]). In this procedure, the stock solution of compounds (4, 5 or 6) (1 mg/mL) was diluted to a final concentration of 2, 4, 6, 8, and 10 μg/mL in methanol. A 1.0 mL of methanolic solution of DPPH (concentration, 0.3 mmol/l) was added to 3.0 mL of the test sample solution of different concentrations. The mixture was shaken and left at room temperature for 30 min, then the absorbance was measured at 517 *nm* using a UV-1800 spectrophotometer and compared with that of the control. The capability to scavenge the DPPH radical was calculated using the following equation:

[% inhibition = 

[(A_control _-A_Sample_) / A_Control_] × 100]

Where A_Sample_ is the absorbance of the DPPH• in the presence of the tested compound and A_Contro_ is the absorbance of the DPPH• in the absence of the tested compound (control). The scavenging activity was expressed as IC_50_ value, which is defined as the concentration (μg/ml) of compound required for scavenging of DPPH radicals by 50 %. IC_50_ values were determined by linear regression analysis using at least five different concentrations in triplicates (Table 2(Tab. 2)).

### Computational methods

#### In silico screening

The *in silico* study was carried out as previously described (Alam et al., 2012[3]). The energy-minimized ligands (compounds **4**-**6**) were drawn with ChemDraw Ultra (2D and 3D) and LigandScout. Discovery studio (Accelrys, 2011[1]), MVD (Thomsen and Christensen, 2006[26]), and Ligand scout (Wolber et al., 2007[29]) were used to determine molecular docking, energy profile of ligand-receptor interactions, and structure activity-relationship, respectively. The coordinates of compounds (**4**-**6**) were also checked using a program for generating molecular topologies (PRODRG). The three-dimensional structures of target protein act as a receptor (PDB ID: 3OEO) were retrieved from the protein data bank. All the heteroatoms associated with protiens including water molecules, bound ligands and any co-crystallized solvent were removed from the PDB file and the missing assignments like proper bonds, bond orders, hybridization and charges were assigned using the Molegro Virtual Docker.

#### FMOs and physicochemical properties

The frontier molecular orbitals (FMOs) and physicochemical properties play a vital role in generating and escalation of bioactivity of natural as well as synthetic compounds (Alam et al., 2011[4]; Alam and Lee, 2015[2]). Highest Occupied Molecular Orbital (HOMO) and Lowest Unoccupied Molecular Orbital (LUMO) are important parameter; they both are called the frontier orbitals (FMOs) which were calculated using GAMESS interface as well as WebMO Job Manager by using the basic level of theory and visualized by using Avogadro software. The frontier orbitals determine the way interact with other species in biological as well as in chemical systems using their outer most orbitals through making a plate form of accepting and donor system. Therefore, the energy of the HOMO and LUMO are directly related to the ionization potential and the electron affinity, respectively (Table 3(Tab. 3)). 

The physicochemical parameters of the synthesized compounds were determined applying the following programs: CS ChemBioDraw Ultra, MarvinSketch and Molinspiration software. CLogP (octanol/ water partition coefficient) is most frequently utilized and most important physico-chemical parameter in QSAR studies. This descriptor is calculated by the methodology developed by Molinspiration as a sum of fragment-based contributions and correction factors. TPSA is calculated based on the methodology published by (Ertl et al., 2000[13]) as a sum of fragment contributions, oxygen and nitrogen centered polar fragments are considered. The maps of molecular lipophilicity potential (MLP) and TPSA were viewed in Molinspiration available online and ChemAxon software.

#### Field points of Steroidal oxime-ethers

The structure of the compound **6** was drawn using Chem3D Pro 12.0 software and subsequently structure was energetically minimized using MOPAC with MM2 and saved MDL molfile (∗mol). Field points were performed to identify the surface properties around the compounds using TorchLite software of Cresset-group. A representative field point pattern is shown by colours and sizes**.** Larger field points are indicative of the stronger points of potential interaction.

## Results and Discussion

### Chemistry

Reaction of the steroidal oxime, cholest-5-en-7-one oxime (**1**), and its analogues, 3-acetoxy (**2**) and 3-chloro (**3**), with chloroethylamine hydrochloride using basic alumina as a solid support under microwave irradiation yielded 7-(2′-aminoethoxyimino)-cholest-5-ene (**4**), 3β-acetoxy-7-(2′-aminoethoxyimino)-cholest-5-ene (**5**), and 3β-chloro-7-(2′-aminoethoxyimino)-cholest-5-ene (**6**), respectively, in appreciable yields (Figure 1(Fig. 1)). The reaction time varied from 2 to 3 min. Comparative results of previously reported compounds (**5** and **6**) in terms of yield and reaction period are given. All spectral and analytical data for compounds **5** and **6** were identical with those previously reported (Shamsuzzaman et al., 2003[22]). Comparison of conventional and microwave syntheses of **4**-**6**: (1) conventional procedure reported (Shamsuzzaman et al., 2003[22]); compound **5**, yield: 90 %, time: 1 h, compound **6**, yield: 85 %, time: 1 h, (2) microwave procedure; compound **4**, yield: 85 %, time: 2.5 min, compound **5**, yield: 93 %, time: 2.0 min, compound **6**, yield: 90 %, time: 3.0 min, respectively. Regarding the steroidal oxime-ether derivatives (**4-6**), formation of an oxime-ether linkage was supported by IR, NMR (^1^H and ^13^C), mass spectral, and C, H, N elemental analyses. All compounds exhibited satisfactory elemental analyses (C, H, N) within ± 0.4 % of theoretical values. Elemental analysis of compound **4 **confirmed a molecular formula of C_29_H_50_N_2_O. IR spectroscopy provided significant evidence for the formation of an oxime-ether linkage. Characteristic absorption bands at 3475, 1665, 1400 and 1235 cm^-1 ^attributed to NH_2_, C=N, N-O, and C-N stretching, respectively, suggesting oxime-ether linkage formation. Similarly, ^1^H NMR spectroscopy confirmed a singlet for a vinylic proton at 5.91 ppm. The downfield chemical shift of H-6 in compound **4** at 5.91 ppm confirmed the *Z*-configuration of the oxime derivatives and *Z*-configuration of its starting materials (**1**-**3**) (Cui et al., 2009[11]). A distorted multiplet for two protons (NCH_2_) at 3.3 ppm, a distorted triplet for two protons (OCH_2_) at 3.80 ppm, and a singlet for two amino (NH_2_) protons at 2.7 ppm further supports the formation of compound **4**_. _Other important peaks were observed at 1.11, 0.91, 0.86, and 0.71 ppm, indicating the presence of angular and side chain methyl protons in the steroidal skeleton. ^13^C NMR spectroscopy of compound **4** also demonstrated characteristic signals at 169.1, 153.3, 113.9, 69.3, and 55.9 ppm for C=N, C-5, C-6, OCH_2_, and NCH_2_ groups, respectively. Remaining carbon atoms of steroidal oxime-ethers were also found to resonate in the expected region. Moreover, mass spectrum of compound **4** further established its formation and displayed ion peaks for respective fragments. Molecular ion peak was observed at *m/z* 443.0 [M+1], which was in agreement with its molecular formula, C_29_H_50_N_2_O, and other notable peaks included *m/z* 382 (M-OCH_2_CH_2_NH_2_) and 329 (M-C_8_H_17_). Hence, the compound can be characterized as 7-(2'-aminoethoxyimino)-cholest-5-ene (**4**), which was first synthesized in the present study. Similarly, the structures of compounds **5 **and **6** were corroborated on the basis of their elemental analyses and spectral data. The physicochemical data (IR, ^1^H NMR and melting points) of compounds (**5** and **6**) were in close proximity to the literature data (Shamsuzzaman et al., 2003[22]). 

### Pharmacology

#### Antibacterial activity

**Figure 2:**
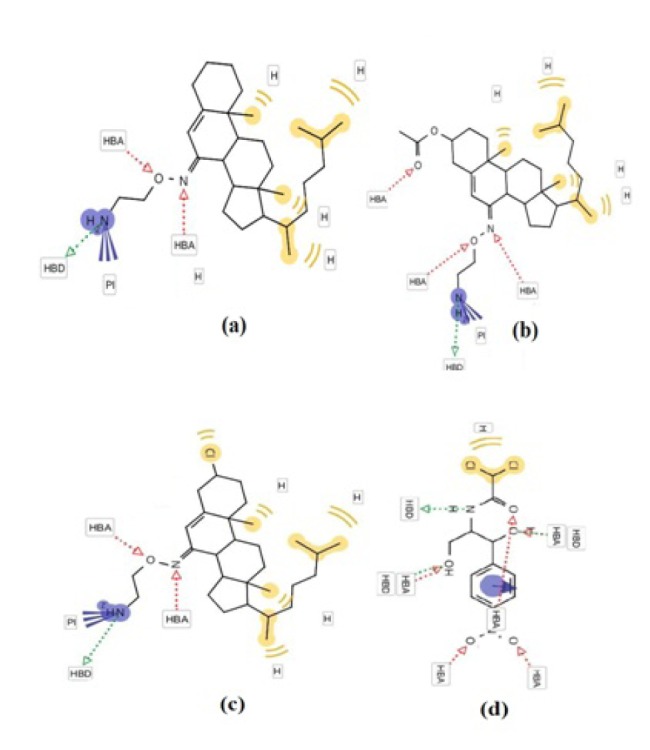
The interaction profile of 4-6 (a-c) and reference drug (d) showing the most probable binding sites. The binding site of compound 6 (c) was found to be in close proximity to the binding site of chloramphenicol (d).

**Figure 3:**
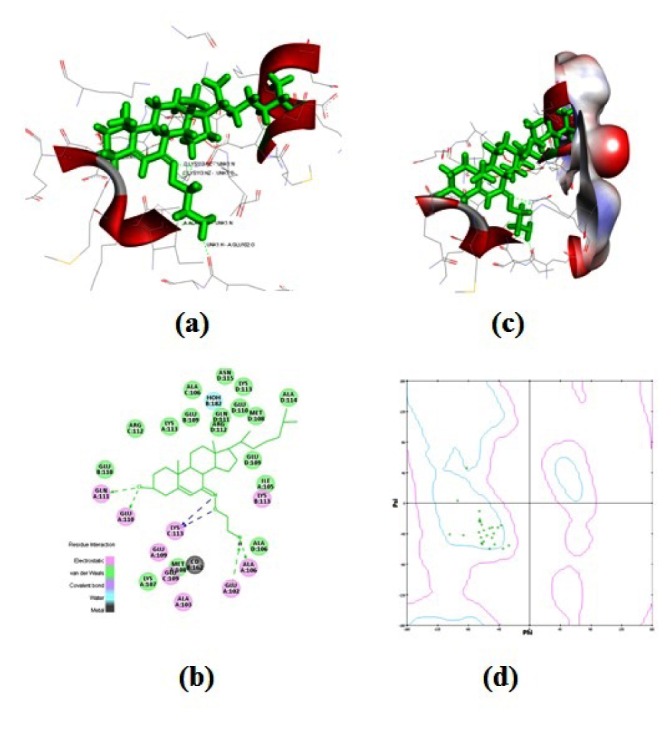
Structural details of ligand-protien interactions (a) docking of compound 6 into the active site of the receptor (3OEO pdb), (b) receptor surface around compound 6, (c) pharmacophore models of protein-ligand interactions, and (d) Ramachandran plot showing pairs of (*Φ*, *Ψ*) angles for all active amino acid residues.

The in vitro antibacterial activities of compounds **4**-**6** were tested against Bacillus subtilis, Streptococcus pyogenes, Staphylococcus aureus, Pseudomonas aeruginosa, Klebsiella pneumoniae and Escherichia coli by disc diffusion method in the concentration of 10 μg/ mL. The growth inhibitory potentials of the tested compounds were compared with that of chloramphenicol, a standard drug (Khan et al., 2012[17]). Compounds were found to inhibit pathogens B. subtilis, S. pyogenes, S. aureus, P. aeruginosa, K. pneumoniae and E. coli. The antimicrobial activity of compound **5** was the highest among the three compounds (Table 1(Tab. 1)), and it exhibited similar inhibition against some strains as chloramphenicol. The structure-activity relationship (SAR) of the steroidal oxime-ether derivatives was determined by introducing acetoxy- or chloro- group at the 3-position of the cholestane ring of the products. In the antimicrobial results, the compounds having electron-withdrawing groups in their cholestane moieties at position 3 (**5** and **6**) (Figure 2(Fig. 2) and 3(Fig. 3)) displayed potent inhibitory activities, whereas non-substituted oxime-ether derivative **4** exhibited poor activity against most of the strain. This behaviour could be attributed to the interactions between the compounds and the receptor (amino acids). The poor activity of compound **4** can also be due to the lack of availability of lone pair to form hydrogen bonds (hydrogen bond acceptor HBA and hydrogen bond donor HBD) with the receptor in compliance with the concept of drug-likeness.

#### Antioxidant activity

The synthesized compounds were screened for their antioxidant potential by employing the *in vitro* DPPH free radical scavenging assay. The free radical scavenging activity of the steroidal oxime-ethers were tested through their capacity to quench the DPPH using ascorbic acid as a standard. The summary of potencies for the antioxidant activity of **4**-**6** to the ascorbic drug is shown in Table 2**(Tab. 2).** Practically, all the synthesized compounds were found to be less potent than that of the reference. Among the synthesized compounds, compound **6** exhibited a slightly better antioxidant activity than compounds **4** and **5**. The structure-activity relationships in this series can be correlated on the basis of the substituent's nature. The presence of the electronegative group on A ring of steroidal skeleton at 3-position facilitate the reduction of DPPH supplying a drift of electron density in order to reduce the DPPH radical. Moreover, the better activity of compound **6** can be interpreted taking the nature of frontier orbitals. A molecule with high values of HOMO energy and dipole moment values insinuates that the molecule is a good electron-donor and compound **6 **has the highest value of HOMO (-258.85 eV) energy and dipole moment (2.33502 Db), therefore, suggests the possibility of high electron donating ability. The main atomic contributions for HOMO and LUMO are shown in Table 3(Tab. 3).

### Computational studies

#### Structure activity relationship (SAR)

The relationship between the chemical structure and biological activities can be ascertained on the basis of 3D coordinate structure under the category of structure activity relationship (SAR). This is further secured on analyzing their biological activity, particularly by screening the synthesized compounds for their plausible mechanism antibacterial activity. The study of SAR provides the structural information, including positions and the nature of the functional groups present in the skeleton and their behavior in a targeted biological mechanism in the organism. Though, it is difficult to draw any conclusions about the molecular events from the structure activity relations. The structure activity relations with compounds having substituent at the 3-position of steroidal skeleton have been attempted to simplify the coordination between the chemical structures and their biological behaviors. The SAR of steroidal oxime-ethers were made by introducing the substituent at 3-position in the steroidal moiety of the synthesized compounds. The antibacterial data as mentioned Table 1(Tab. 1) reveals that the antibacterial activity was more affected by the compounds having additional functional groups in the cholestane skeleton at position 3 (**5** and **6**) (Figure 1(Fig. 1)) while the non-substituted oxime-ethers **4 **shown poor activity against most of the strain. The variation of biological activity of the same carbon skeleton with different functional groups may be attributed to lack of proper interaction between compounds and receptor. The poor activity of **4** among series may also due to the unavailability of lone pair to make a certain number of hydrogen bonds with a receptor in compliance with the concept of drug-likeness in Figure 2(Fig. 2) showing the probable binding sites including reference drug.

#### In silico study

In order to verify the remarkable antibacterial activities of **4-6**, *in silico* screening of these compounds were also performed. *In silico* screening was carried out in order to distinguish the nature and extent of the interactions of compounds **4-6** with the pathogenic receptor (3OEO pdb). The binding site of compound **6** was found to be in close proximity to the binding site of chloramphenicol, as evident in Figures 2(Fig. 2) and 3(Fig. 3). Based on the molecular docking studies and probable site of ligand, it was found that 

**Figure 4:**
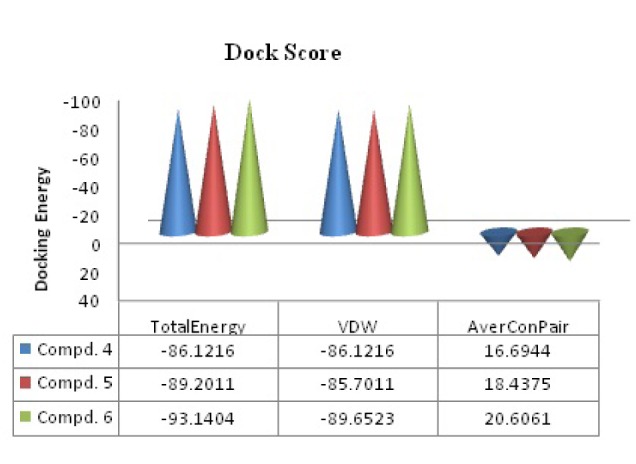
Predictable binding affinities of compounds (4-6) based on docked poses within the active site of target protein (PDB: 3OEO). (Where VDW= van der Waals' interaction energy and average Conpair). *In vitro* studies correlated well with the results of above docking studies.

A:ALA106:N-:UNK1:N, 

A:GLU110:N-:UNK1:Cl, Arg266:NH1, 

A:GLN111:N-:UNK1:Cl, 

C:LYS113:NZ-:UNK1:O, 

C:LYS113:NZ-:UNK1:N, 

and :UNK1:H - A:GLU102:O 

residues (where UNK = ligand or molecule; Figure 2a(Fig. 2) and 2c(Fig. 2)) interacted with compound **6** in proper orientation and with comparatively high frequency. Since the docking score is representative of the binding affinity of ligand to receptor. The binding scores from iGEMDOCK (Yang et al., 2004[30]) for compound **6** with reference to standard drug chloramphenicol were also calculated. GEMDOCK Method: Compound **6**; -93.14, binding affinity (Figure 4(Fig. 4)): Chloramphenicol; -111.5, binding affinity. The affinity of pathogenic protein with the compound **6** has been shown to be the highest and the total energy stabilization (Fitness = vdW + Hbond + Elec) is -93.14, of which -89.652 is the contribution of van der Waal interactions and the remaining energy is the stabilization resulting due to H-bonds. It can be inferred from Table 1(Tab. 1) and dock score as shown in Figure 4(Fig. 4) that *in vitro* studies correlated well with the results of the docking studies.

The above scores clearly depicts that the binding energy of compound **6** is close to the standard drug, Chloramphenicol implying a stronger interaction between compound **6** and the protein 3OEO pdb. The value of these non-covalent interactions is a good approximate in developing effective drug candidates as these values also predict the strength of protein-drug interaction. Moreover, Ramachandran plot (Figure 2d(Fig. 2)) (Gunda et al., 2012[15]; Emsley et al., 2010[12]) demonstrates the stable stereochemistry and overall geometry of the phi-psi angle of the amino acid residues. The Ramachandran plot was studied in order to ascertain the stereochemical quality of the protein model, 3OEO pdb. This plot was drawn after the *in silico* docking of the protein. The Ramachandran plot exhibited the statistical probability of active binding sites of protein in terms of favored and disallowed regions. It shows 91.6 % favored and 0.6 % disallowed regions respectively, indicating it to be a good quality model. 

**Figure 5:**
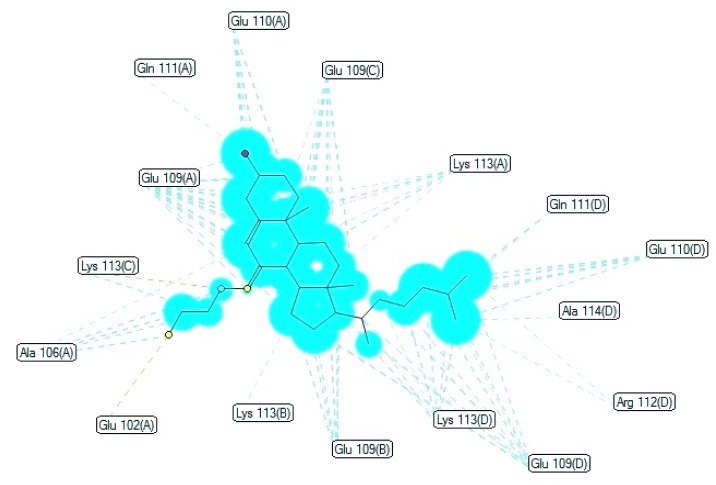
Depiction of hydrogen bonding and hydrophobic interactions overlay with receptor

Ligand map (Figure 5(Fig. 5)) was used to understand the in-depth interaction between docked compound and the active site region of the pathogenic receptor. Number of noncovalent interactions such as hydrophobic, hydrogen bonds and van der Waals forces and their pattern were matched with the help of diagrams automatically generated by Molegro Molecular Viewer software. This software can compute and analyze the way ligands interact with macromolecules. Ligand map provides a set of divergent intuitive features and are also in useful in knowing the secondary interaction patterns after successful operations.

#### FMOs and physicochemical properties

**Figure 6:**
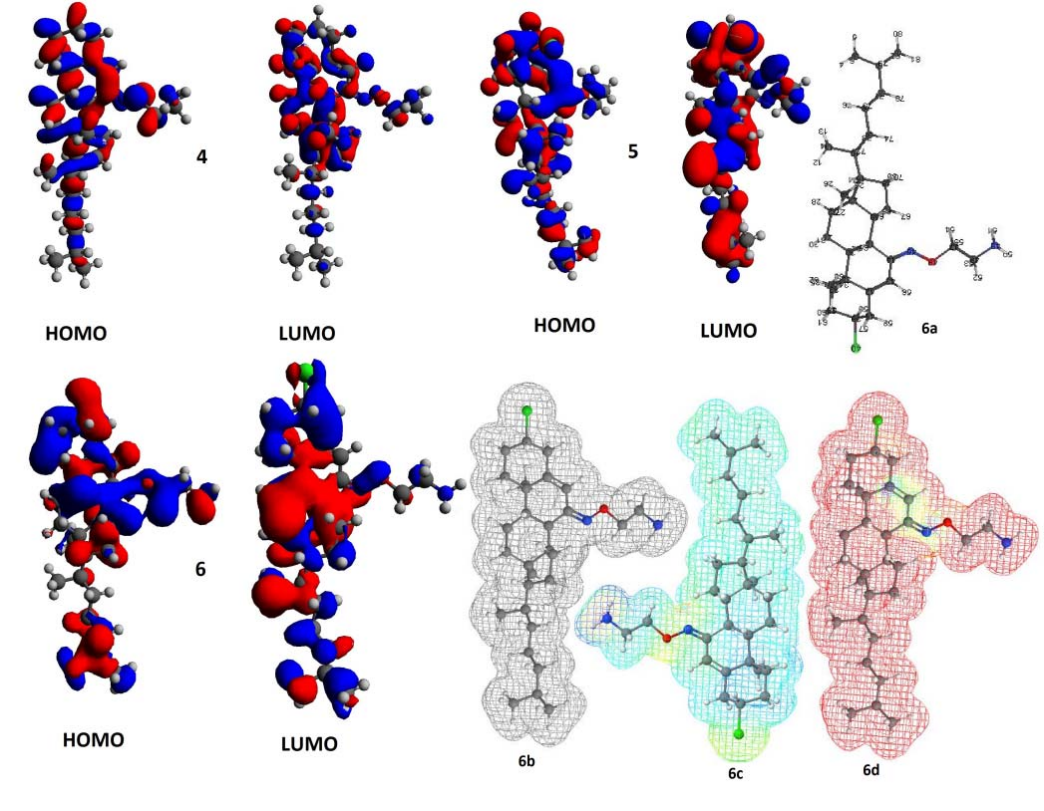
Computational study; the HOMO and LUMO isosurface for compounds 4-6 (Different surface colours show opposite signs of wave function along with geometry optimized structure 6a, electrostatic potential 6b, electron density 6c, and radical frontier density surfaces 6d (All the structures have been adjusted in such a way, they occupy minimum space).

To explain the antibacterial assay of eco-friendly way of the synthesized steroidal oxime-ethers, the quantum chemical and physicochemical calculation were performed by DFT at B3LYP/6-31G and basic set of theory using Gamess interface as well as WebMO Pro Job Manager for calculation and Avogadro software for visualization and analysis. Quantum-chemical and two important physicochemical parameters are shown in Table 3(Tab. 3). The calculated values of eigen function directly related to the energy of compound **6** (-4592816.33kJ/mol) is lower than that of compounds **4** (-3400714.12 kJ/mol) and **5** (-3987879.35kJ/mol), explaining the more thermodynamic stability of **6**. The HOMO-LUMO energy difference of **6** is higher than that of **5** and less than that of **4**, however the total energy of **6** is lower than that of **4** and **5** derivatives. The energies of the FMOs are important properties in several chemical and pharmacological strategies. The differences in the energies of FMOs establishes properties of the system and give information on the electron donating and accepting character of a compound bridging between HOMO-LUMO systems (Figure 6(Fig. 6)). The energy of the highest occupied molecular orbital (E_HOMO_) measures the electron donating character of a compound and the energy of the lowest unoccupied molecular orbital (E_LUMO_) measures its electron accepting character. From these definitions, the greater the E_HOMO_, the greater will be the electron donating capability, the smaller the E_LUMO_, the smaller will be the resistance to accept electrons. For the active compounds, the E_HOMO_ must bring negative values, whereas the less active/inactive compounds must have positive values as shown in Table 3(Tab. 3). Compounds showing the less activity in the pharmacological process are more efficient electron donor compounds than that of the active ones. Though, the inactive compounds may interact through a charge transfer mechanism with some compounds before reaching the biological receptor. Moreover, dipole moments as shown in Table 3(Tab. 3) are highly correlated with the activity followed by molecular weight. It is observed that the higher the dipole moment and the higher the activity.

The lipophilic character of molecules, expressed as *logP*, plays an important role in determining molecular reactivity in a biological process and depends on the two important factors i.e. hydrophobicity and polarity. These factors of the molecules facilitate to penetrate the cellular membrane consisting of a number of heterogeneous phases or irreversibly damage the cellular membrane. 

**Figure 7:**
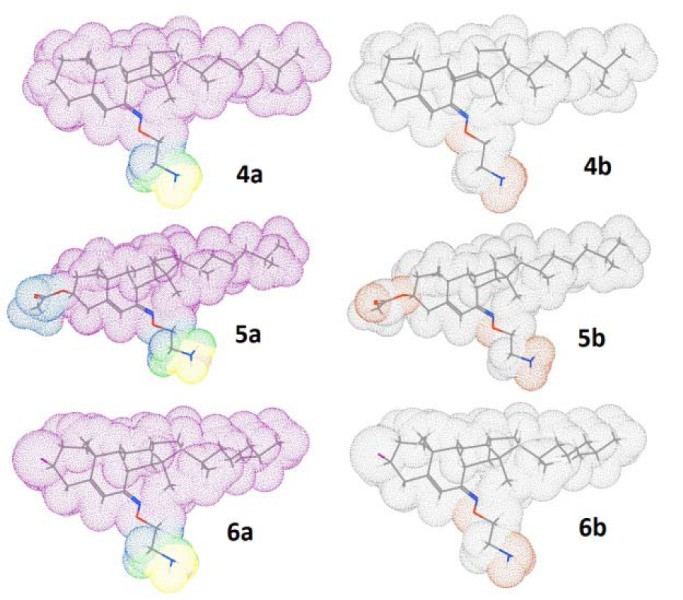
3D surface of the compounds (4-6) with molecular lipophilicity maps, 4a-6a (left side) and polar surface pockets, 4b-6b (right side) showing the most lipophilic area (pink color), intermediate lipophilic area (green color), most hydrophobic area (blue color), nonpolar area (gray white color) and polar area (red color).

Figure 7(Fig. 7) shows the molecular lipophilicity potential map (MLP) of the synthesized compounds (**4**-**6**) suggesting that the compound with a chloro- group at 3-position of the steroidal skeleton is more lipophilic (7.587) than that of its analogues (4 & 5; 7.334 & 6.753). In the present study, the *log P* of the most active compounds is higher under the certain limits than those of less active compounds in biological processes. In general, higher the value of the calculated *log P* favors for the activity. However, there are some exceptions to this rule and some compounds do not obey the trend of the rule of five, but still participate in biological system and to kill or check the propagation of the pathogenic strain (Coates and Hu, 2008[9]). Partition coefficient or Indeed, *Log P *is an important parameter used in rational drug design to measure molecular hydrophobicity. Hydrophilic/ lipophilic nature of drug molecule affects drug absorption, bioavailability, drug receptor interactions, metabolism of molecules, as well as their toxicity. The Log P values of derivatives were found to be in the range of 6.753-7.587 and are a clear violation of Lipinski's rule of five and often a *logP* value of > 5 is considered as an upper limit of desired lipophilicity. In this series, none of the compounds **(4-6)** fulfilled Lipinski's rule as their Log P score was above **5 and 6** suggesting these compounds are highly lipophilic with very poor aqueous solubility. Moreover, it is found that the molecular volume also plays important role in SAR studies to model molecular properties and biological activity. It was also observed that the active compounds **5** and **6** have higher molecular volume as compared with **4**. Molecular polar surface area (PSA) is closely related to the hydrogen bonding potential of a molecule and is a very useful parameter for prediction of drug transport properties. From the Figure 7(Fig. 7) and Table 3**(Tab. 3),** all the compounds (**4**-**6**) were found in the range of 47.621 and 73.926 and below the limit, that is, 160 A˚ in respect of PSA, which showed that molecules are fulfilling the optimal requirement for drug absorption.

**Figure 8:**
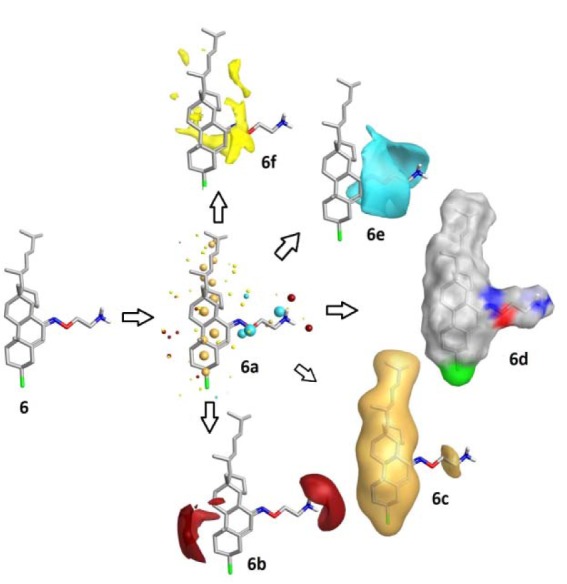
Figure 8: Field patterns and physicochemical properties; compound 6 showing the size of the point indicates the potential strength of the interaction 6a; red: positive field points 6b (like to interact with negatives/H-bond acceptors on a protein); gold/orange: hydrophobic field points 6c (describe regions with high polarizability/hydrophobicity); white smoke: solvent-accessible molecular surface 6d; blue: negative field points 6e (like to interact with positives/H-bond donors on a protein) and yellow: van der Waals surface field points 6f (describing possible surface/ vdW interactions)

The surface properties of the active compound **6** among three have been described using reliable TorchLite software of Cresset-group. With the help of Field points, the activity and properties of compound **6** on the surface of the compound may be determined using four molecular fieldss i.e. the electrostatic (positive and negative), steric and hydrophobic properties (Nami et., 2012[19]) and shown in Figure 8(Fig. 8). These fields elaborate the molecular interactions between ligand and receptor target. Moreover, these surface phenomena are also applied to draw the compound in specific conformation and has been depicted in Figure 7(Fig. 7).

## Conclusion

The present study reports the eco-friendly synthesis of steroidal oxime-ethers on alumina as a solid support by employing microwave irradiation. This route is convenient, reproducible, and affords oxime-ethers in a short reaction time and with improved yields in a solvent-free environment. These steroidal oxime-ethers may be further explored for their possible antimicrobial and anticancer activities. Computational studies can suggest the useful information for understanding the inhibition mechanism with the divergent interpretations. Molecular docking studies may elucidate the mechanisms of the steroid-oxime ether as effective biologically active agent. Hence, these compounds may be regarded as a potential antimicrobial and antioxidant candidate for further investigation.

## Figures and Tables

**Table 1:**
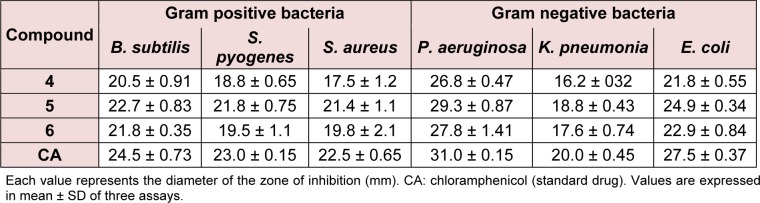
Antimicrobial activities of steroidal oxime-ethers 4-6 using disc diffusion method

**Table 2:**
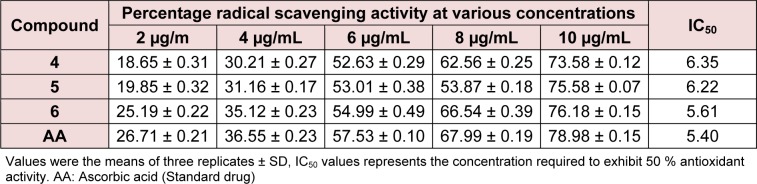
Table 2: Antioxidant activity of synthesized compounds (4-6)

**Table 3:**
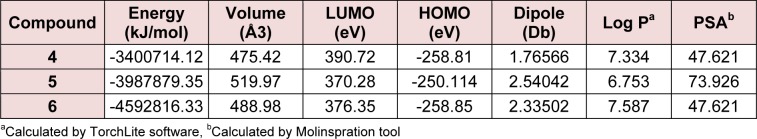
Comparison of quantum-chemical and physicochemical properties of steroidal oximes (4-6).
